# Different patterns of *Toxoplasma gondii* infection epidemiology in the general population, animal contact workers, and blood donors in southeastern China between 2019 and 2023: a cross-sectional study

**DOI:** 10.1051/parasite/2025061

**Published:** 2025-10-22

**Authors:** Xiaoxiao Wang, Wei Ruan, Wenjie Xu, Hualiang Chen, Jiaqi Zhang, Xuan Zhang, Kegen Yu, Qiaoyi Lu, Zhen Wang, Jimin Sun

**Affiliations:** Zhejiang Provincial Center for Disease Control and Prevention 310051 Hangzhou PR China

**Keywords:** Animal contact workers, Blood donors, Cross-sectional study, Occupational exposure, Seroprevalence

## Abstract

There are estimated to be over one billion human infections of *Toxoplasma gondii* worldwide. However, significant spatial heterogeneity exists across countries and regions. For this study, a total of 2,943 participants were enrolled between 2019 and 2023 in Zhejiang Province, southeastern China including 519 animal contact workers, 1,722 people from the general population, and 702 blood donors. Anti-*T. gondii* IgG and IgM in sera were assessed using an enzyme-linked immunosorbent assay. The overall seroprevalence of IgG and IgM was 4.08% and 0.41%, respectively. IgG positivity was highest in the (50, 60] years age group (5.47%, 29/530), while IgM was found in the (60, 70] years age group (1.21%, 3/247). The general population showed the lowest IgG seroprevalence (1.68%) compared to animal contact workers (10.40%) and blood donors (5.27%) (*p <* 0.001). Patterns of *T. gondii* IgG prevalence varied by participant type. Increasing seroprevalence with age was observed among animal contact workers, indicating a cumulative effect, while frequency was highest in the (50, 60] and (30, 40] age groups in the general population and blood donors, respectively. Animal contact workers with two types of animal exposure had higher IgG positivity rates (13.16%) than those with one type (8.50%). Occupational exposure to cattle was associated with the highest frequency of IgG (12.69%), followed by pigs (9.69%), and sheep (8.85%). This study provides critical insights into the epidemiological characteristics of *T. gondii* infections across distinct population groups in Eastern China.

## Abstract (fr)

On estime à plus d’un milliard le nombre d’infections humaines à *Toxoplasma gondii* dans le monde. Cependant, une hétérogénéité spatiale significative existe entre les pays et les régions. Au total, 2 943 participants ont été recrutés dans cette étude entre 2019 et 2023 dans la province du Zhejiang, dont 519 travailleurs en contact avec les animaux, 1 722 personnes de la population générale et 702 donneurs de sang. Les IgG et IgM anti-*T. gondii* dans le sérum ont été évaluées par dosage immuno-enzymatique. La séroprévalence globale des IgG et IgM était respectivement de 4,08 % et 0,45 %. La positivité des IgG était maximale dans le groupe d’âge (50-60] (5,47 %, 29/530), tandis que les IgM ont été trouvées surtout dans le groupe d’âge (60-70] (1,21 %, 3/247). La population générale a montré la plus faible séroprévalence des IgG (1,68 %) par rapport aux travailleurs en contact avec les animaux (10,40 %) et aux donneurs de sang (5,27 %) (*p* < 0,001). Les schémas de prévalence des IgG contre *T. gondii* variaient selon le type de participant. Une séroprévalence croissante avec l’âge a été observée parmi les travailleurs en contact avec les animaux, indiquant un effet cumulatif, tandis que la fréquence était maximale respectivement dans les groupes d’âge (50-60] dans la population générale et (30-40] chez les donneurs de sang. Les travailleurs en contact avec les animaux avec deux types d’exposition aux animaux avaient des taux de positivité des IgG plus élevés (13,16 %) que ceux avec un seul type (8,50 %). L’exposition professionnelle aux bovins était associée à la fréquence la plus élevée d’IgG (12,69 %), suivis de l’exposition aux porcs (9,69 %) et aux moutons (8,85 %). Cette étude fournit des informations essentielles sur les caractéristiques épidémiologiques des infections à *T. gondii* dans différents groupes de population en Chine orientale.

## Background

Toxoplasmosis, one of the most common parasitic infections, is caused by the obligate intracellular protozoan *Toxoplasma gondii*. Although the only definitive hosts of *T. gondii* are felids, there is a wide variety of intermediate host species, especially mammals, birds, livestock, and humans [11]. Humans are mainly infected by ingesting raw or undercooked meat, water, vegetables, and fruits contaminated with sporulated oocysts [14]. The severity of toxoplasmosis in humans varies, ranging from asymptomatic infections to severe ocular and neurological lesions. While primary infections in adults are mostly asymptomatic, infections in immunodeficient or immunocompetent patients may cause fatal toxoplasmic encephalitis, myocarditis, and pneumonitis [23]. Additionally, infections during pregnancy pose a risk of mother-to-child transmission or serious damage to the developing fetus, including long-term disabilities, stillbirth, or fetal death [39].

Globally, over one billion people are estimated to be infected with *T. gondii* [17]. However, significant spatial heterogeneity has been reported across various countries and regions. Infection prevalence ranges from 30% to 90% in Central America, South America, and continental Europe [30, 42], compared to 8% to 22% in the United States [2, 12]. The epidemiology of *T. gondii* infection is influenced by factors such as climate [29, 43], cultural practices [38], and food habits [44]. High seroprevalence rates have been reported in regions where consumption of raw or undercooked meat is common, and where contact with contaminated soil or water is prevalent [15, 37]. Additionally, socioeconomic factors [5], such as income per capita and human development index, can influence the prevalence of *T. gondii* infections within populations [5, 34].

Diagnostic methods available for detecting *T. gondii* infection in humans primarily involve histopathology, immunohistochemistry, molecular methods, and indirect (serological) tests [23, 27]. Molecular methods, including polymerase chain reaction (PCR), offer a more specific approach by identifying *T. gondii* DNA in blood, tissue, or other bodily fluids, which is particularly useful for detecting active infections or in immunocompromised patients [19, 27]. Serological tests such as enzyme-linked immunosorbent assay (ELISA) and indirect immunofluorescence assay (IFA), which detect antibodies against the parasite, are generally highly sensitive and are widely used because of their accessibility and simplicity [21, 25].

Understanding the prevalence or seroprevalence of *T. gondii* infection among different population groups is crucial for identifying high-risk populations and implementing targeted public health interventions. Despite numerous epidemiological studies in various countries or regions, updated serosurveys focusing on *T. gondii* in southeast China remain limited. This study aimed to investigate the seroprevalence of *T. gondii* infection in the general population, occupational groups with close contact with animals, and blood donors in Zhejiang Province, southeastern China. By employing serological methods, the study sought to elucidate epidemiological patterns and inform disease management policies.

## Methods

### Ethics approval

The study was approved by the ethics review board of the Zhejiang Provincial Center for Disease Control and Prevention (No. 2021-004-01, January 27th, 2021).

### Study sites

This study was conducted between 2019 and 2023 and included five counties in Zhejiang Province: Deqing, Dongyang, Yiwu, Ninghai, and Pujiang. Dongyang, Yiwu, and Pujiang are located in central Zhejiang, while Deqing and Ninghai are in the northern and eastern parts of the province, respectively.

### Participants

Three groups of participants were recruited: the general population, animal contact workers, and blood donors. Each year, 100–200 participants undergoing routine physical examinations at town-level hospitals in Dongyang, Yiwu, Ninghai, and Pujiang were included in the general population. Animal contacts – workers occupationally exposed to animals, animal breeders, meat processing and sales personnel, and veterinarians – were surveyed in Deqing, Dongyang, Ninghai, and Pujiang. Specific details regarding the type of animal contact were collected from each participant. Blood donors were recruited from county-level blood centers in Dongyang, Yiwu, Ninghai, and Pujiang. Data on age, sex, and risk factors associated with *T. gondii* infection of animal contact workers, such as exposure type and animals involved, were collected from participants.

### Sample collection

Approximately 2 mL of venous blood was collected from each participant. Specimens were stored at 4 °C and transported to a local center for disease control and prevention within 24 h. Additionally, all blood samples were separated into sera, red blood cells, and white blood cells within 24 h of blood collection and stored at −20 °C until analysis.

### Serologic tests

Anti-*T. gondii* IgG and IgM were detected in serum samples using commercial ELISA kits (NovaTec Immunodiagnostica GmbH, Dietzenbach, German), following the manufacturer’s instructions. Positive, negative, and blank controls were included in each test batch.

### Data analysis

Data were organized using Microsoft Office Excel 2021 (Microsoft Corporation, Redmond, WA, USA). All statistical analyses were performed using R Project, version 4.2.3 (R Foundation for Statistical Computing, Vienna, Austria). A map of the study site location and prevalence of antibodies at each site was generated using ArcGIS, version 10.1 (Esri, Redlands, CA, USA). Categorical variables were analyzed using the chi-squared test, with Fisher’s exact test applied when appropriate. Logistic regression was used to analyze the risk factors for *T. gondii* infection. Statistical significance was set at a *p*-value of < 0.05.

## Results

### General information

**Figure 1:**
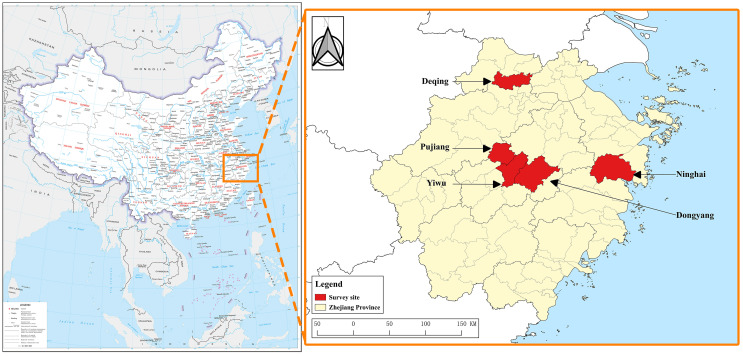
Location of study sites, produced based on the standard map service website of the Ministry of Natural Resources of China GS (2022) 4314, with no modification of the base map boundary.

A total of 2,943 participants were enrolled from five study sites between 2019 and 2023, including 519 animal contact workers, 1,722 people from the general population, and 702 blood donors (Fig. 1; see Supplementary Table 1). The median age of the study population was 44 years (range, 1–88). Of the total participants, 1,513 were male and 1,430 were female, with similar median ages (women, 44 years; men, 42 years).

### Overall prevalence of *T. gondii* infections

Considerable spatial diversity was observed in the seroprevalence of *T. gondii*. The highest IgG seroprevalence was found in Ninghai (6.00%, 50/833) and Deqing (4.40%, 4/91), followed by Dongyang (4.53%, 52/1,148). Pujiang had the lowest positivity (1.34%, 9/671) (see Supplementary Table 2). Anti‐*T. gondii* IgG and IgM antibodies were detected in 120 and 12 participants, respectively, yielding an apparent IgG seroprevalence of 4.08% (120/2,943) and a small IgM seroprevalence of 0.41% (12/2,943). Notably, four samples tested positive for both IgG and IgM (Table 1). The prevalence for middle age (30–50] was 4.76% for IgG and 0.40% for IgM.

**Table 1:** Overall seroprevalence of *T. gondii* in Zhejiang Province, 2019 to 2023.

Year	No. of participants	IgG positive		IgM positive	
		No. positive	Prevalence (%)	No. positive	Prevalence (%)
2019	91	4	4.40	1	1.10
2020	200	5	2.50	0	0
2021	624	16	2.56	2	0.32
2022	681	30	4.41	3	0.44
2023	1,347	65	4.83	6	0.45
Total	2,943	120	4.08	12	0.41

**Figure S1.**
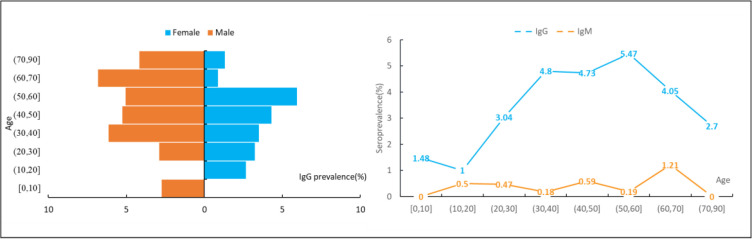
Seroprevalence of *T. gondii* by gender and age in all participants.

An increasing trend was observed in participants aged ≤ 60 years, while a decline was observed in those aged > 60 years (Table 2). IgG positivity rate by age showed a similar pattern, with overall seroprevalence of *T. gondii* highest in the (50, 60] age group (5.47%, 29/530)*.* The highest prevalence of positive IgM was found in the (60, 70] age group (1.21%, 3/247), while the prevalence in other groups was below 1% (see Supplementary Figure 1).

**Table 2:** Overall seroprevalence of *T. gondii* by age group.

Age	No. of participants	IgG Positive		IgM positive	
		No. positive	Prevalence (%)	No. positive	Prevalence (%)
[0,10]	134	2	1.49	0	0
(10, 20]	200	2	1	1	0.5
(20, 30]	428	13	3.04	2	0.47
(30, 40]	563	27	4.8	1	0.18
(40, 50]	677	32	4.73	4	0.59
(50, 60]	530	29	5.47	1	0.19
(60, 70]	247	10	4.05	3	1.21
(70, 90]	148	4	2.7	0	0
NA	16*	1	6.25	0	0
Total	2,943	120	4.08	12	0.41

* The ages of 16 participants were not available.

Differing patterns were observed between men and women. Men exhibited higher IgG positivity rates (4.56%, 69/1,513) compared to women (3.57%, 51/1,430) (χ^2^ = 1.857, *p* = 0.173). Furthermore, the positivity rate in men was highest in the (60, 70] age group, whereas it was highest in the (50, 60] age group for women (see Supplementary Figure 1).

### Seroprevalence of *T. gondii* in the general population

**Figure 2:**
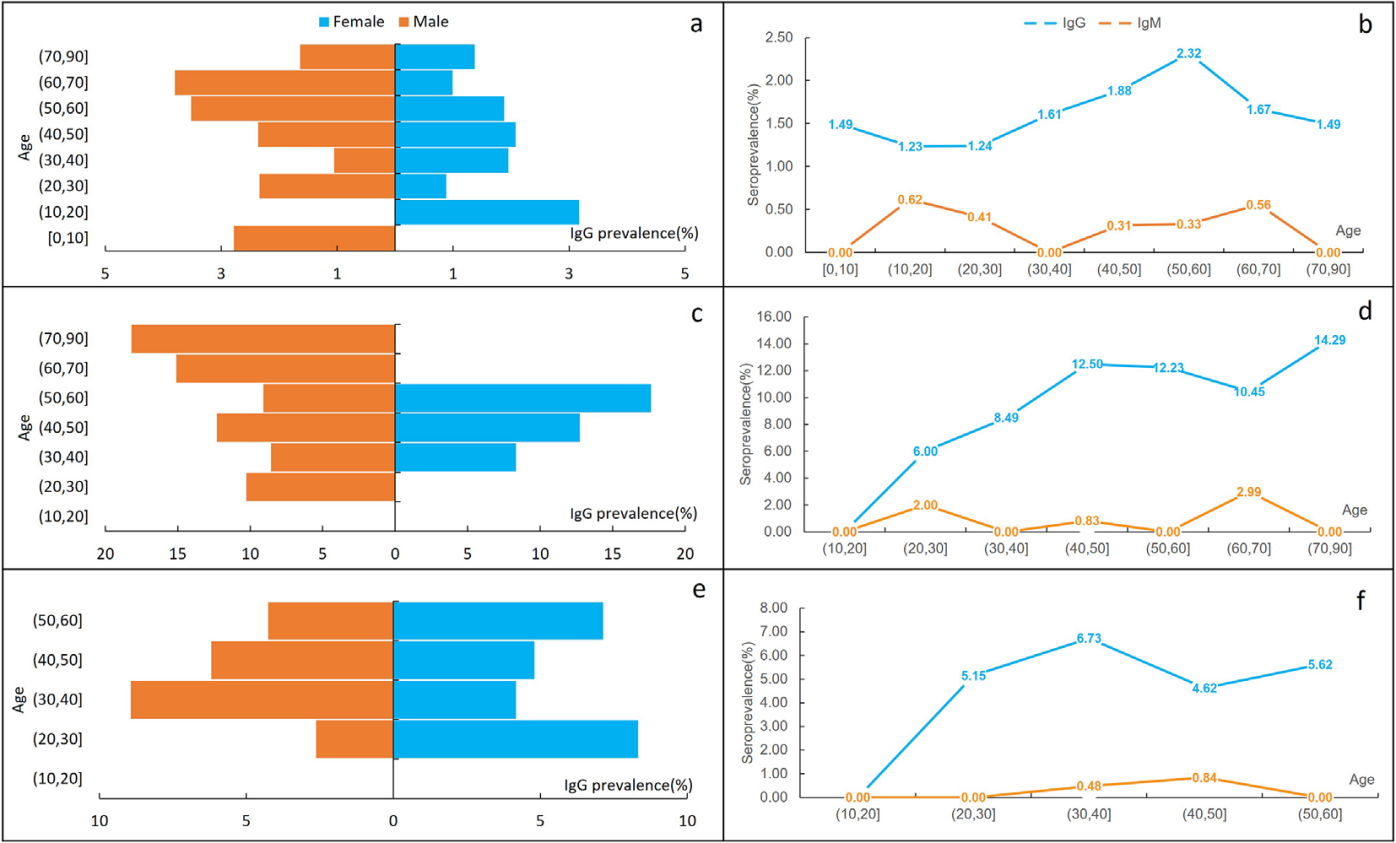
Seroprevalence of *Toxoplasma gondii* (IgG) by gender and age in the general population, animal contact workers, and blood donors: (a and b, general population; c and d, animal contact workers; e and f, blood donors).

The general population consisted of 803 women and 919 men with a median age of 43 years (range, 1–88). The overall IgG prevalence in the general population was 1.68% (29/1,722), with higher positivity rates (> 2.00%) observed among farmers, freelancers, healthcare workers, and individuals in privately owned businesses (Table 3). While men had higher IgG prevalence rates (1.74%, 14/803) than women (1.63%, 15/919), the difference was not statistically significant (χ^2^ = 0.031, *p* = 0.860). The IgG seroprevalence increased with age and was highest in the (50, 60] age group (2.32%, 7/302) before declining to 1.67% (3/180) in the (60, 70] age group and 1.49% (2/134) in the > 70 age group. In contrast, IgM positivity remained low (< 1.0%) across all age groups (Table 4, Fig. 2).

**Table 3:** *T. gondii* seroprevalence by participants types.

Participants Types	No. of participants	IgG positive		IgM positive		Statistical value
		No. positive	Prevalence (%)	No. positive	Prevalence (%)	
Animal contact workers	519	54	10.40	4	0.77	IgG: χ^2^ = 80.897, *p* < 0.001
Blood donors	702	37	5.27	3	0.43	IgM: *χ*^*2*^ = 2.275, *p* = 0.321
General population	1,722	29	1.68	5	0.29	
Freelancer	81	2	2.47	1	1.23	*p* = 0.468
Healthcare workers	27	1	3.70	0	0	
Individually owned business	72	2	2.78	0	0	
Farmer	550	10	1.82	2	0.36	
Worker	260	5	1.92	0	0	
Children below 6 yrs and students	216	3	1.39	0	0	
Business and service	371	4	1.08	2	0.54	
Retiree	41	0	0	0	0	
White-collar class	64	0	0	0	0	
NA	40*	2	5.00	0	0	
Total	2,943	120	4.08	12	0.41	

* Occupations of 40 participants were not available.

**Table 4:** IgG prevalence of *T. gondii* in the general population, animal contact workers, and blood donors by age.

Age	General population		Animal contacts		Blood donors	
	No. positive	Prevalence (%)	No. positive	Prevalence (%)	No. positive	Prevalence (%)
[0, 10]	2	1.49	/	/	/	/
(10, 20]	2	1.23	0	0	0	0
(20, 30]	3	1.24	3	6.00	7	5.15
(30, 40]	4	1.61	9	8.49	14	6.73
(40, 50]	6	1.88	15	12.50	11	4.62
(50, 60]	7	2.32	17	12.23	5	5.62
(60, 70]	3	1.67	7	10.45	/	/
(70, 90]	2	1.49	2	14.29	/	/
NA	/	/	1	6.25	/	/
Total	29	1.68	54	10.40	37	5.27

### Seroprevalence of *T. gondii* in animal contact workers

The median age of the animal contact workers was 49 years (range, 18–83) and included 341 men and 178 women. A high seroprevalence was observed among animal contact workers, with the majority being IgG positive (10.40%, 54/519), while IgM prevalence remained low at 0.77% (4/519) (Table 3). Two participants tested positive for both IgG and IgM. The overall positivity rate was similar between males (10.26%, 35/341) and females (10.67%, 19/178). The positivity frequency increased with age, and was also consistently higher than the general population and blood donors, with the highest IgG prevalence found in the (70, 90] age group at 14.29% (2/14) (Table 4, Fig. 2). Multivariate analysis showed that the infection risk of animal contact workers is statistically higher than that of the general population (OR = 6.202, 95% CI: 3.793–10.140) (Table 5).

**Table 5:** Risk factors for *T. gondii* infection among participants.

Variables	*β*	SE	Wald χ^2^	OR	95% CI	*p*-Value
Gender	−0.039	0.196	0.040	0.961	0.655–1.412	0.841
Type						
General population	Ref.					
Animal contact workers	1.825	0.251	52.937	6.202	3.793–10.140	<0.001
Blood donor	1.147	0.271	17.952	3.147	1.852–5.349	<0.001
Age groups						
0 – 10	Ref.					
11 – 20	−0.819	1.014	0.653	0.441	0.060–3.215	0.419
21 – 30	−0.088	0.786	0.013	0.916	0.196–4.275	0.911
31 – 40	0.204	0.764	0.071	1.226	0.274–5.484	0.790
41 – 50	0.228	0.760	0.090	1.255	0.283–5.565	0.765
51 – 60	0.373	0.759	0.242	1.453	0.328–6.435	0.623
61 – 70	0.177	0.802	0.049	1.193	0.248–5.741	0.826
71 – 90	0.232	0.883	0.069	1.261	0.224–7.110	0.793

Regarding specific types of animal exposure, workers with two types of animal exposure showed a higher IgG positivity rate (13.16%) compared to those with a single type (8.50%), although this difference was not statistically significant (χ^2^ = 2.911, *p* = 0.088). The IgG positivity proportions for slaughterers, breeders, and cleaners were 9.09% (15/165), 9.26% (10/108), and 9.09 % (1/11), respectively. Workers with two exposure types exhibited higher seroprevalence: breeders + slaughterers (20.00%), sales + processing (13.07%), and sales + slaughterers (12.73%). No positive IgG or IgM was detected in milkers, veterinarians, or managers (Table 6).

**Table 6:** *T. gondii* seroprevalence by exposure types of animal contacts.

Exposure types	No. of participants	IgG positive		IgM positive	
		No. positive	Prevalence (%)	No. positive	Prevalence (%)
Two types	**213**	**28**	**13.16**	**0**	**0**
Breeders + slaughters	5	1	20.00	0	0
Sales + processing	153	20	13.07	1	0.65
Sales + slaughters	55	7	12.73	2	3.64
One type	**306**	**26**	**8.50**	**0**	**0**
Slaughter	165	15	9.09	0	0
Breeder	108	10	9.26	1	0.93
Cleaner	11	1	9.09	0	0
Management	7	0	0	0	0
Milker	7	0	0	0	0
Veterinary	8	0	0	0	0
Total	519	54	10.40	4	0.77

* Differences between two types and one type.

**Figure 3:**
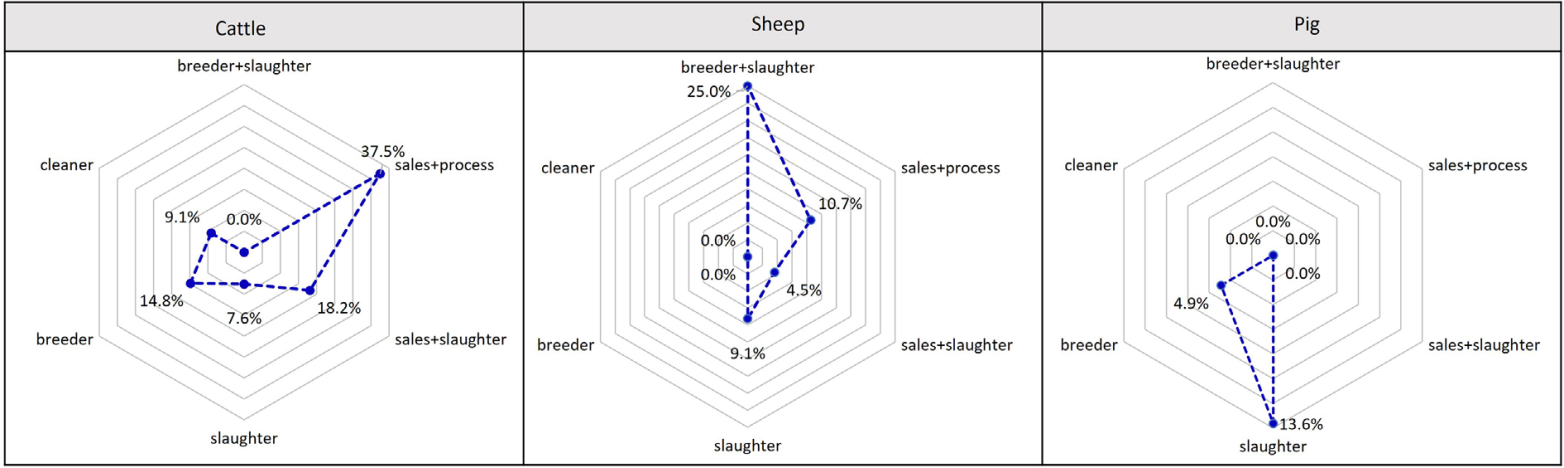
Interactive effect between exposure types and exposed animals in animal contact workers.

The highest IgG frequency was found among workers occupationally exposed to cattle (12.69%), followed by those exposed to sheep (9.69%) and pigs (8.85%) (Table 7) (χ^2^ = 1.4322, *p* = 0.489). Further analysis of interactive effects between exposure types and exposed animals revealed that among cattle contact workers, “sales + processing” had the highest seroprevalence at 37.5%, followed by “sales + slaughterer” (18.18%) (*p* = 0.048). For sheep contacts, “breeder + slaughterer” (25.00%) and “sales + processing” (10.69%) had higher seroprevalence (*p* = 0.547). Among pig contact workers, slaughterers showed a higher positivity rate than breeders (χ^2^ = 1.261, *p* = 0.261) (Fig. 3).

**Table 7:** *T. gondii* seroprevalence by exposed animals of animal contact persons.

Exposed animals	No. of participants	IgG positive		IgM positive	
		No. positive	Prevalence (%)	No. positive	Prevalence (%)
Cattle	197	25	12.69	2	1.01
Pig	113	10	8.85	1	0.88
Sheep	196	19	9.69	1	0.51
Dog	5	0	0	0	0
Sheep + cattle	5	0	0	0	0
Pig + cattle	1	0	0	0	0
NA	2*	0	0	0	0
Total	519	54	10.40	4	0.77

* Exposed animals from two participants were not available. NA, not available.

### Seroprevalence of *T. gondii* in blood donors

The 702 blood donors enrolled in the study were aged 18–59 years (median, 39 years), with 369 (52.56%) being male. Nearly 6% of blood donors were found to be *T. gondii* seropositive (5.56%, 39/702), most of whom were IgG positive (5.27%, 37/702) (Table 3). Three participants were IgM positive (0.43%, 3/702). A similar IgG positivity rate was observed between males (5.42%, 20/369) and females (5.11%, 17/333). IgG positivity was not observed in the (10, 20] age group but increased to 5.15% in the (20, 30] age group and plateaued at 5%–6% in the 20–60 age group.

**Figure 4:**
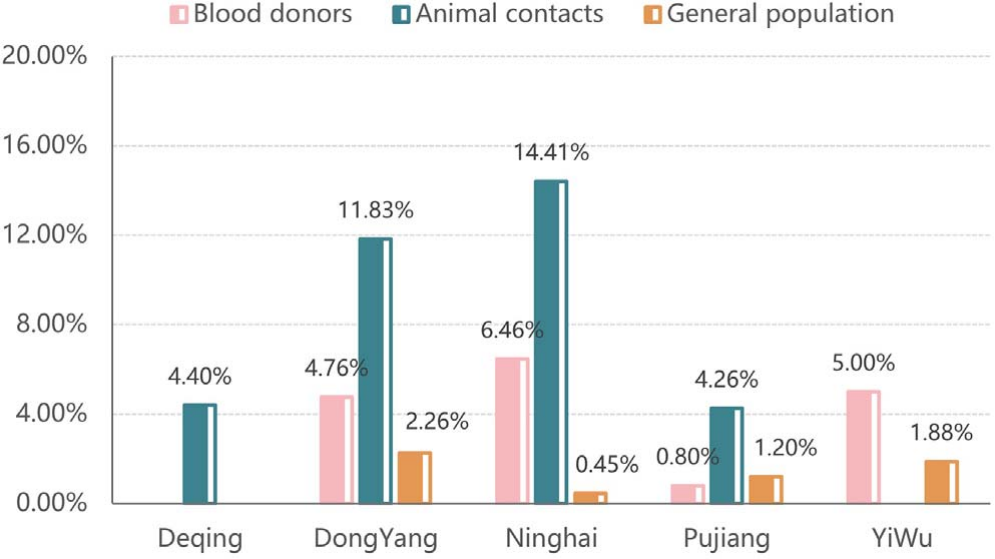
Seroprevalence of *Toxoplasma gondii* (IgG) by participants types in study sites.

Of note, the IgG level was higher in blood donors than in the general population in each age group (20, 30], (30, 40], (40, 50], and (50, 60], with statistically significance, despite younger age distribution. The odds ratio for *T. gondii* infection between blood donors and general population was statistically significant (OR = 3.147, 95% CI: 1.852–5.349) (Table 5). There was a statistically significant difference among study sites (*p* = 0.043), with the lowest rate reported in Pujiang (0.80%, 1/125) and rates ≥ 5.0% in Yiwu (5.0%, 2/40), Ninghai (6.46%, 32/495), and Dongyang (4.76%, 2/42) (Fig. 4).

## Discussion

Measurement of IgM and IgG seroprevalence offers an efficient way to assess the incidence of *T. gondii* infections in populations. Our serosurvey revealed a low seropositivity rate (IgG, 4.08%; IgM, 0.41%) for toxoplasmosis in the general population in Zhejiang province, southeastern China. This rate is considerably lower than those reported in other countries, such as Germany (IgG, 49.08%) [42], Italy (IgG, 28.2%; IgM, 1.3%) [32], France (IgG, 55.38%) [7], the United Kingdom (IgG, 16.07%) [28], and the United States (IgG, 10.8%–31%) [12, 20]. Several factors likely contribute to the low prevalence in our study. First, there has been a low prevalence of *T. gondii* among food animals (pooled seroprevalence, 15.3%) in China in recent years [44]. Second, raw or undercooked meat is seldom consumed in Zhejiang province. Third, economic and social development in recent years has greatly improved individual hygiene habits, thereby reducing exposure to the parasite. Similar to findings for Europe [8], seroprevalence increased with age, with a maximum in the (50, 60] age group (5.47%), likely due to lifelong exposure. Men showed marginally higher IgG rates than women (4.56% *vs.* 3.57%), although the difference was not statistically significant (*p* = 0.173), contrasting with European data showing no consistent gender difference. Furthermore, the pattern of *T. gondii* IgG antibody prevalence in our serological survey differed among the general population, animal contact workers, and blood donors. Specifically, the positivity rate was highest in the (50, 60] and (30, 40] age groups in the general population and blood donors, respectively, before declining in older age groups. In contrast, a cumulative effect with age was observed among animal contact workers. The observed association between IgG prevalence and age in the general population contrasts with previous studies, which commonly report higher seroprevalence in older age groups [7, 32]. This discrepancy may reflect geographical heterogeneity between countries or regions. Other possible explanations include survivor bias in older individuals, which is common in cross-sectional studies, as well as antibody attenuation of *T. gondii* in older individuals. While IgM has a short duration (median 12.8 months) with high variability [13], IgG is reported to persist for years or indefinitely [13]. Cohort studies are needed to determine the decline pattern of IgG in the general population.

It is reported that animal contact workers have a higher prevalence of *T. gondii* infection owing to their higher exposure opportunities, especially in Africa (84.0%) [9], followed by Europe (72.8%) [3], the Middle East (46.1%) [16], and the Americas (44.4%) [1]. The overall positivity rate of *T. gondii* infection in the occupationally exposed population in China is relatively lower (5.0%–18.0%). However, conflicting data exist regarding the association between animal contact and *T. gondii* infection risk [9, 35, 40]. A study from Canada found no increased risk in individuals exposed to animals [35]. Another study from India reported a low seroprevalence of toxoplasmosis among veterinary personnel [40]. These conflicting findings may be related to various factors, such as different regions, social environments, survey time, and study design. Furthermore, discrepancies in seroprevalence rates between studies may also stem from variations among ELISA kits, which lack standardized antigens, protocols, and cutoff thresholds, making direct comparisons challenging. Our study confirmed that animal contact workers were more likely to have IgG seropositivity than the general population (10.40% *vs.* 1.68%), demonstrating an association between animal exposure and *T. gondii* infection. This association is further supported by higher seropositivity rates observed among individuals with exposure to two or more types of animals, emphasizing the role of occupational exposure. Additionally, seroprevalence in animal contact workers increased with advancing age, consistent with studies from Finland [36] and India [10], highlighting the cumulative risk of animal exposure. Based on these findings, routine surveillance and countermeasures are suggested for high-risk groups to prevent and minimize the incidence of toxoplasmosis, such as using personal protective equipment and promoting hand hygiene practices.

While sheep/goats are classical intermediate hosts, our data suggest that cattle-associated work deserves greater scrutiny [38]. Potential mechanisms include environmental contamination from cattle farm/factory facilitating oocyst transmission, or cattle handlers in our study area may have prolonged exposure to contaminated soil/water, a known reservoir for oocysts. In addition, cattle workers’ heightened exposure may also stem from combined occupational and behavioral factors, such as inadequate hygiene. Our results reinforce the need for species-specific protective measures alongside dietary education.

Screening for *T. gondii* in blood banks is not mandatory globally [6]. However, *T. gondii* transmission from blood donors to recipients has raised concerns because of the parasite’s ability to remain viable in blood for nearly 2 months, which increases the possibility of transmission by blood or component transfusions [41]. Blood recipients, who are often immunocompromised or immunosuppressed, are particularly vulnerable to serious clinical consequences from *T. gondii* infection. Seropositivity rates among blood donors vary widely across different regions (10–80%) or even within a country [4, 10, 18]. A geographical disparity in *T. gondii* antibody prevalence has been reported in Mexico [4]. Furthermore, a meta-analysis in Iran, with a sample size of 4,538, reported seroprevalence of 18.3%–56.4% among blood donors [26]. Lupu *et al.* also found that nearly half of blood donors were *T. gondii* antibody-positive in Romania [24]. Additionally, high seroprevalence rates were found in blood donors from Brazil [31], Portugal [33], and Tunisia [22]. In contrast, our study found a low IgG positivity rate of 5.27%, which was much lower than those reported in other countries, but comparable with the 6.26% prevalence shown by a systematic review from 1986 to 2017 in China. The relatively low overall positivity of *T. gondii* antibodies in our study is likely because the majority of the population in Zhejiang province consumes well-cooked food. However, the IgG prevalence in blood donors in our study was significantly higher than that in the general population (5.27% *vs.* 1.68%) (χ^2^ = 80.897, *p* < 0.001), indicating that *T. gondii* infections are more common among blood donors in Zhejiang province. This is inconsistent with European trends [8], where blood donors typically reflect lower-risk groups. IgG positivity rates of blood donors by occupation were all higher than those of the general population (farmers: 3.47% *vs.* 1.82%; freelancers: 7.06% *vs.* 2.47%; healthcare workers: 7.04% *vs.* 3.70%; workers: 5.00% *vs.* 1.92%; white-collar: 6.63% *vs.* 0%), although patterns between them were comparable. These findings underscore potential donor-specific risk factors (*e.g.*, lifestyle, regional exposures) and highlight the public health imperative for enhanced *T. gondii* screening in blood donors.

This study has several limitations. First, specimens from the general population were collected from individuals attending routine physical examinations rather than a random sample, which may introduce selection bias. Second, IgG avidity was not tested to discriminate between acute and chronic toxoplasmosis. Third, information on risk factors was not collected to explore the possible reasons for high seroprevalence in animal contact workers and blood donors, which warrants further investigations.

## Conclusions

This study demonstrated the epidemiological characteristics of *T. gondii* infection in the general population, animal contact workers, and blood donors in southeastern China. Variable patterns of *T. gondii* antibody distribution were revealed among the three participant types. Additionally, a cumulative risk was observed with age in animal contacts, and individuals with more animal exposure types exhibited higher seroprevalence. These findings highlight the importance of enhanced surveillance and targeted awareness programs for high-risk groups, particularly animal contact workers, to mitigate potential exposure.

## Data Availability

The dataset used and/or analyzed during the current study is available from the corresponding author on reasonable request.

## Supplementary material

*Table S1*. Sample numbers at different study sites. *Table S2*. Seroprevalence of *T. gondii* at different study sites.

The supplementary material of this article is available at https://www.parasite-journal.org/10.1051/parasite/2025061/olm.
