# Supplementary material for: Different patterns of Toxoplasma gondii infection epidemiology in the general population, animal contact workers, and blood donors in southeastern China between 2019 and 2023: a cross-sectional study
Source: Parasite. 2025 Oct 22;32:68. doi: 10.1051/parasite/2025061 (PMC12543297; doi:10.1051/parasite/2025061)
Supplement: Supplementary file 1 — Table S1. Sample numbers at different study sites. [file parasite-32-68-s1.pdf]

Table S1. Sample numbers in different study sites

Year	Study sites				
	Deqing	DongYang	Ninghai	Pujiang	YiWu
2019	91				
2020					200
2021		300			
2022		334		324	
2023		514	833	347	
