# Supplementary material for: Different patterns of Toxoplasma gondii infection epidemiology in the general population, animal contact workers, and blood donors in southeastern China between 2019 and 2023: a cross-sectional study
Source: Parasite. 2025 Oct 22;32:68. doi: 10.1051/parasite/2025061 (PMC12543297; doi:10.1051/parasite/2025061)
Supplement: Supplementary file 2 — Table S2. Seroprevalence of T. gondii at different study sites. [file parasite-32-68-s2.pdf]

Table S2. Seroprevalence of *T. gondii* in different study sites

Sites	No. of participants	IgG positive		IgM positive	
		No.	Prevalence (%)	No.	Prevalence (%)
Deqing	91	4	4.40	1	1.10
DongYang	1148	52	4.53	7	0.61
Ninghai	833	50	6.00	4	0.48
Pujiang	671	9	1.34	0	0
YiWu	200	5	2.50	0	0
Total	2943	120	4.08	12	0.45
